# The healing effect of platelet-rich plasma on xenograft in peri-implant bone defects in rabbits

**DOI:** 10.1186/s40902-016-0061-5

**Published:** 2016-03-24

**Authors:** Wang Peng, Il-kyu Kim, Hyun-young Cho, Ji-Hoon Seo, Dong-Hwan Lee, Jun-Min Jang, Seung-Hoon Park

**Affiliations:** 1 Department of Oral and Maxillofacial Surgery, College of Medicine, Inha University, Incheon, South Korea; 2 Department of Oral and Maxillofacial Surgery, International St. Mary’s Hospital, Catholic Kwandong University College of Medicine, Incheon, South Korea; 3 Department of OMFS, Dentistry, College of Medicine, Inha University, #7-206, 3rd St. Shinheung-dong, Choong-gu, Incheon, 400-711 South Korea

**Keywords:** Bone healing, Dental implant, Platelet-rich plasma, Xenograft, Rabbit

## Abstract

**Background:**

The association of biomaterial combined with repair factor-like platelet-rich plasma (PRP) has prospective values. Bovine-derived xenograft has been identified as an osteoconductive and biocompatible grafting material that provides osseointegration ability. PRP has become a valuable adjunctive agent to promote healing in a lot of dental and oral surgery procedures. However, there are controversies with respect to the regenerative capacity of PRP and the real benefits of its use in bone grafts. The purpose of this study was to assess the influence of PRP combined with xenograft for the repair of peri-implant bone defects.

**Methods:**

Twelve rabbits were used in this study, and the experimental surgery with implant installation was performed simultaneously. Autologous PRP was prepared before the surgical procedure. An intrabony defect (7.0 mm in diameter and 3.0 mm deep) was created in the tibia of each rabbit; then, 24 titanium dental implants (3.0 mm in diameter and 8.5 mm long) were inserted into these osteotomy sites. Thus, a standardized gap (4.0 mm) was established between the surrounding bony walls and the implant surface. The gaps were treated with either xenograft alone (control group) or xenograft combined with PRP (experimental group). After healing for 1, 2, 3, 4, 5, and 6 weeks, the rabbits were sacrificed with an overdose of KCl solution. Two rabbits were killed at each time, and the samples including dental implants and surrounding bone were collected and processed for histological analysis.

**Results:**

More newly formed bone and a better bone healing process were observed in control group. The histomorphometric analysis revealed that the mean percentage of bone-to-implant contact in the control group was significantly higher than that of the experimental group (25.23 vs. 8.16 %; *P* < 0.05, independent-simple *t* test, analysis of variance [ANOVA]).

**Conclusions:**

The results indicate that in the addition of PRP to bovine-derived xenograft in the repair of bone defects around the implant, PRP may delay peri-implant bone healing.

## Background

The use of a dental implant has become a common treatment and an important part of modern dentistry. Immediate implantation into fresh extraction sockets has been recommended as a means to minimize bone loss and shorten the prosthetic treatment time [1]. However, the residual bone defects, between the implant neck and the residual bone walls, may cause cell migration from the connective and epithelial tissue into the defect area, possibly preventing osseointegration [2], and jeopardize the success of immediate implant procedures [3]. For such defects, bone augmentation procedures in combination with the implant placement are necessary.

There is a continuous search for bone substitutes to minimize or avoid the need for autogenous bone grafts. Several materials, synthetically derived or processed from skeletal structures of other species (xenografts), have been used alternative to the autogenous bone harvest. Bovine-derived xenograft has been widely used as a bone graft material due to abundant sources and accessible processing, which can provide an osteoconductive scaffold and has a mineral content comparable to that of human bone allowing it to integrate with the host bone. It is by far the best documented bone substitute material used in combination with guided bone regeneration [4].

For several years, platelet-rich plasma (PRP) has been thought to promote bone healing, in particular, bone grafting material mixed with PRP has been reported to enhance bone formation. Marx et al. [5] found that the mixture of PRP and autogenous bone grafts can increase the rate of osteogenesis and qualitatively enhance bone formation. Moreover, Trisi et al. [6] reported that PRP, adding to a mixture of autogenous bone and Biogran, could improve the new bone formation, with a reduction in the time needed for graft healing and a greater amount of bone formed after only 5 to 6 months. Recently, PRP has become a valuable adjunct in many dental and oral surgery procedures, such as ablative surgical procedures, periodontal plastic surgery, and treatment of infrabony periodontal defects, as well as procedures relating to the placement of osseointegrated implants [7].

PRP can be defined as a volume of autogenous plasma that has a platelet concentration above the baseline, and it is produced by centrifugation of the patient’s own blood. Platelets release multiple wound-healing growth factors and cytokines, including platelet-derived growth factor (PDGF), transforming growth factor β1 and β2 (TGF-β1 and β2), vascular endothelial growth factor (VEGF), platelet-derived endothelial growth factor, basic fibroblast growth factor, and platelet-activating factor-4 [8]. So, PRP is the suspension of growth factors that has been demonstrated to induce healing and regeneration in soft as well as hard tissues [9].

However, there are contradicting reports about its biologic effect on the enhancement of bone healing. Hatakeyama et al. [10] analyzed the bone healing in the calvarial defects of rabbits by using an autogenous graft with or without PRP, and they found that the association of PRP with autogenous bone did not improve the bone healing process. Furthermore, Jensen et al. [11] investigated the effect of PRP on bone regeneration in an allograft using dog models. They demonstrated that the addition of PRP into an allograft has no effect on new bone formation in the graft.

The inconsistency of these results prompted this study on the effect of PRP on bone healing in a xenograft. Therefore, this experiment was designed to assess the influence of PRP used as an adjunct combined with bovine-derived xenograft for the repair of bone defects adjacent to titanium dental implants in rabbits.

## Methods

### Experimental materials

A total of 24 titanium dental implants (Osstem Implant Co., Busan, Korea), 8.5 mm in length and 3 mm in diameter, were used in this study. Collagen membranes (Bioland, Chungnam, Korea), as barrier membrane, were used to cover the entire surgical site. Twelve healthy female New Zealand rabbits, 5–6 months old, weighing between 3.2 and 3.7 kg, were selected as the animal models. Animal selection and management, surgical protocol, and preparation followed routines were approved by the Institutional Animal Care and Use Committee at the College of Medicine, Inha University, Incheon, Korea.

### Preparation of PRP

Based on the method described by Okuda et al. [12], the PRP was prepared by the transfusion laboratory (Inha University Hospital, Incheon, Korea) using a hematology system (Advia 2120i, Siemens, Erlangen, Germany). Briefly, 4 mL of autologous blood withdrawn from each rabbit, using a 23-gauge needle attached to a 5-mL sterilized syringe, then added to a tube (BD Vacutainer®, BD Co., NJ, USA) containing sodium citrate and mixed. The blood was initially centrifuged at 2400 rpm for 10 min to separate the PRP and platelet-poor plasma (PPP) portions from the red blood cell fraction. The PRP and PPP portions were again centrifuged at 3600 rpm for 15 min to separate the PRP from the PPP. The approximate volume of PRP obtained was 0.5 mL, and then platelet counts were performed. In our experimental animals, the numbers of platelets in the whole blood and PRP were 236 × 10^3^/μL and 625 × 10^3^/μL, respectively. So, the concentration of platelets in PRP is at least 2.6 times higher than the baseline value of platelets by using this hematology system. Before application, the PRP was activated with 10 % calcium chloride solution and 1 KU of bovine thrombin (Sigma-Aldrich, St. Louis, MO, USA). After activation, PRP turned into a gel-like substance and mixed with xenograft (Bio-Oss®, Osstem Implant Co., Busan, Korea) in a ratio of 0.5 mL PRP with 0.5 mg of xenograft.

### Surgical procedure

**Fig. 1:**
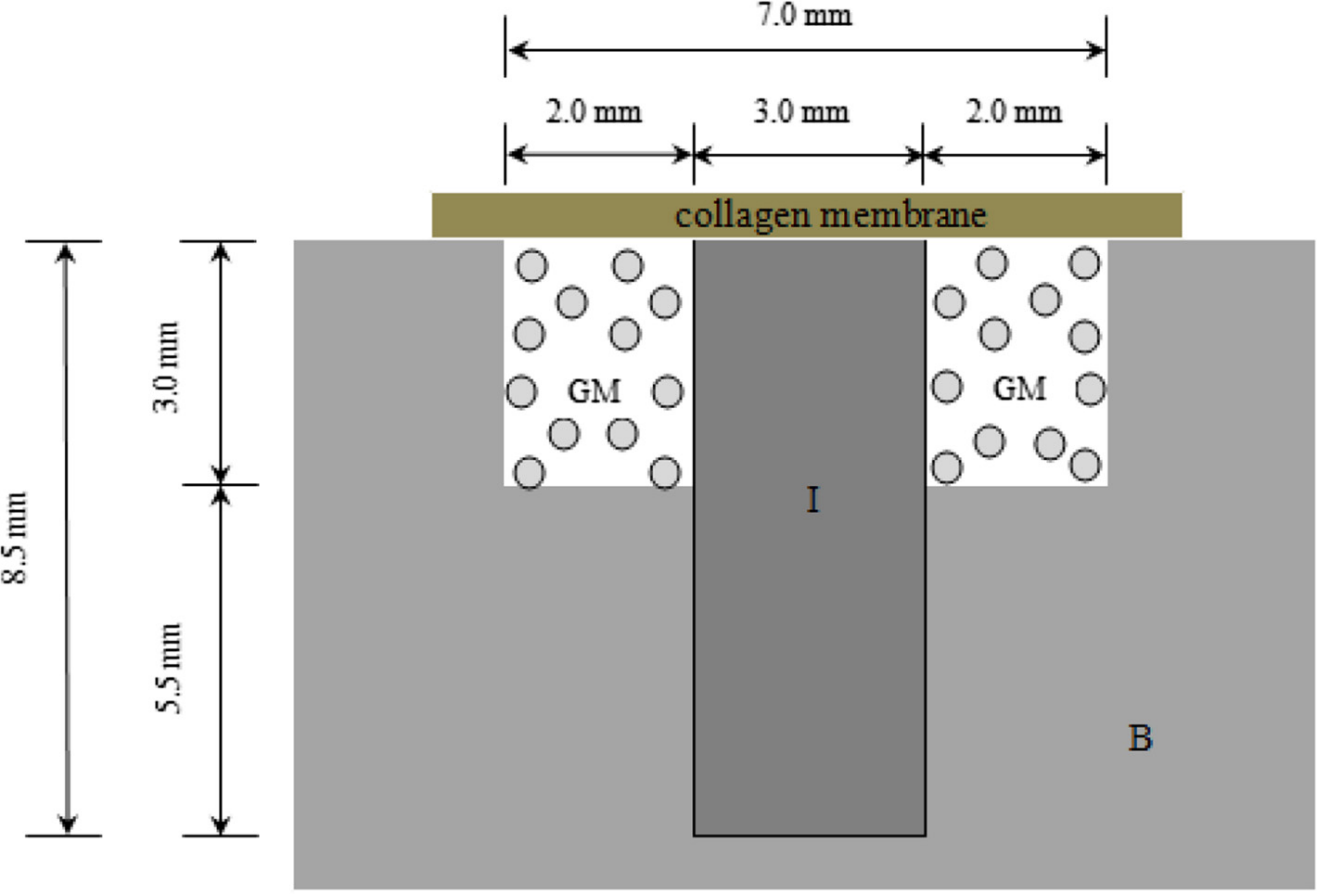
Schematic of experiment. The schematic graph shows the length and diameter of implant fixture around the bone defect

Surgery was conducted on all rabbits under sterile conditions. General anesthesia was induced by intramuscular injection of 5 mg/kg xylazine HCl and 40 mg/kg ketamine HCl, and was maintained by injection of the same mixture with half doses. Then, the incision site was shaved and sterilized. An injection of 2 % lidocaine HCl 1.8 mL containing 1:100,000 epinephrine was used as a local anesthesia at the incision site to reduce subcutaneous hemorrhage. The bone surface of the tibia was exposed by an approximately 3 cm in length incision made on the internal side along the tibial long axis. Before implantation, intrabony defects, approximately 3 mm in depth and going through cortical and cancellous layers, were created at implant recipient site by using a 7.0-mm trephine bur in a low-speed hand piece under continuous sterile saline irrigation. Titanium implants 8.5 mm in length and 3 mm in diameter were then placed through both the defect and the lower cortical bone, so that a standardized gap of 4.0 mm (2.0 mm between the bony walls and each side of implant) was established (Fig. 1). A total of 24 implants were inserted, 1 in each tibia. All the implants were stable at the time of insertion. The bone gaps were treated with the following two treatment modalities: (1) grafting with xenograft alone as the control group and (2) grafting with xenograft mixed with PRP as the experimental group. All experimental areas were covered with absorbable collagen membranes, and then were closed with 4.0 black surgical silks. Postoperatively, all rabbits received an intramuscular injection of 50,000 IU/kg penicillin G (Kunwha Pharmaceutical Co., Seoul, Korea) once daily for 3 days.

### Histological preparation

Twelve rabbits were divided into six groups of two animals with the following healing periods: 1, 2, 3, 4, 5, and 6 weeks. At the end of each designated healing period, two rabbits were sacrificed with an overdose of KCl solution. Implants and surrounding bone from the tibias were removed en bloc and immediately fixed by immersion in 10 % neutral buffered formalin.

Dehydration of the specimens was performed by an increasing and graded series of ethanol (70–100 %), and then embedded in methacrylate-based resin. Resin-infiltration was initiated with a mixture (1/1) of ethanol/Technovit 7200 VLC (Heraeus Kulzer, Wehrheim Germany) for 4 days, followed by infiltration with pure Technovit 7200 VLC for an additional 1 week.

The entire dehydration and infiltration process usually required 2 weeks; the nondecalcifed specimens of infiltration step was performed under vacuum chamber systems depending on the size and substance of the tissue and whether infiltration was performed under a vacuum.

For embedding, the specimen was positioned flush against the bottom of an embedding mold and held in place with a drop of plastic fixation medium. Embedding medium was then added in sufficient volume to surround the specimen. The polymerization of the embedding takes place in a photopolymerization unit (EXAKT Apparatebau, Norderstedt, Germany) with exposure to daylight for 2 h and to ultraviolet light for 10 h.

Polymerized blocks were affixed to the vacuum head of the EXAKT macrocutter, and sections were “sawed” to a thickness of approximately 100 μm. These sections were then ground and polished on the EXAKT microgrinder to a thickness of 15 μm, mounted on microscope slides, stained with hematoxylin and eosin (hematoxylin for 10 min, washed in tap water, and 2 min in eosin, rinsed 3 % acid alcohol), and coverslipped.

The bone healing patterns were observed under a light microscope (BX50; Olympus, Tokyo, Japan).

### Histomorphometry

**Fig. 2:**
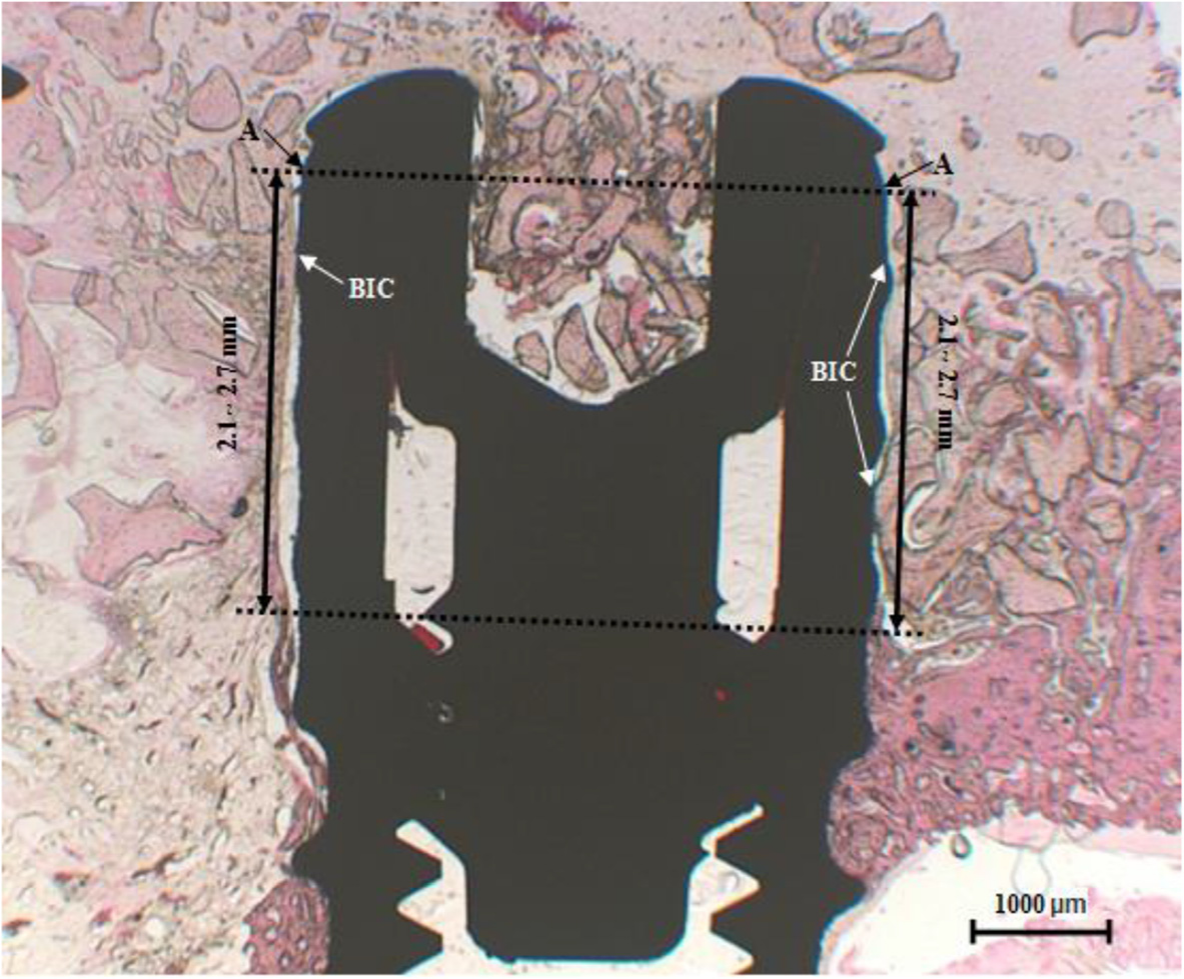
Bone-to-implant contact. The histological micrograph shows the position subjected to BIC measurement

After conventional light-microscopical examination, histomorphometric measurements were made using an automated two-image analysis system Image-Pro Plus (Image-Pro Plus, Media Cybernetics, Silver Spring, MD) and IMT i-Solution Lite (IMT i-Solution Lite ver 8.1, IMT i- Solution. Inc, Canada). After taking a ×12.5 enlarged photograph of the whole tissue specimen, each part required for measurement was measured by ×50 and ×100 magnifications, and various images were taken from one specimen with no overlapping. Bone-to-implant contact, as well as bone healing, defined as the linear length of bone surface in direct contact with implant divided by the implant perimeter starting from the most coronal surface-treated position down to a range of 2.1- to 2.7-mm distance along the implant surface (Fig. 2), was then calculated.

### Statistical analysis

Histomorphometric differences between the two active treatments were analyzed using an independent simple *t* test and analysis of variance (ANOVA). The significance level was set at *P* < 0.05.

## Results

### Histologic findings

**Fig. 3:**
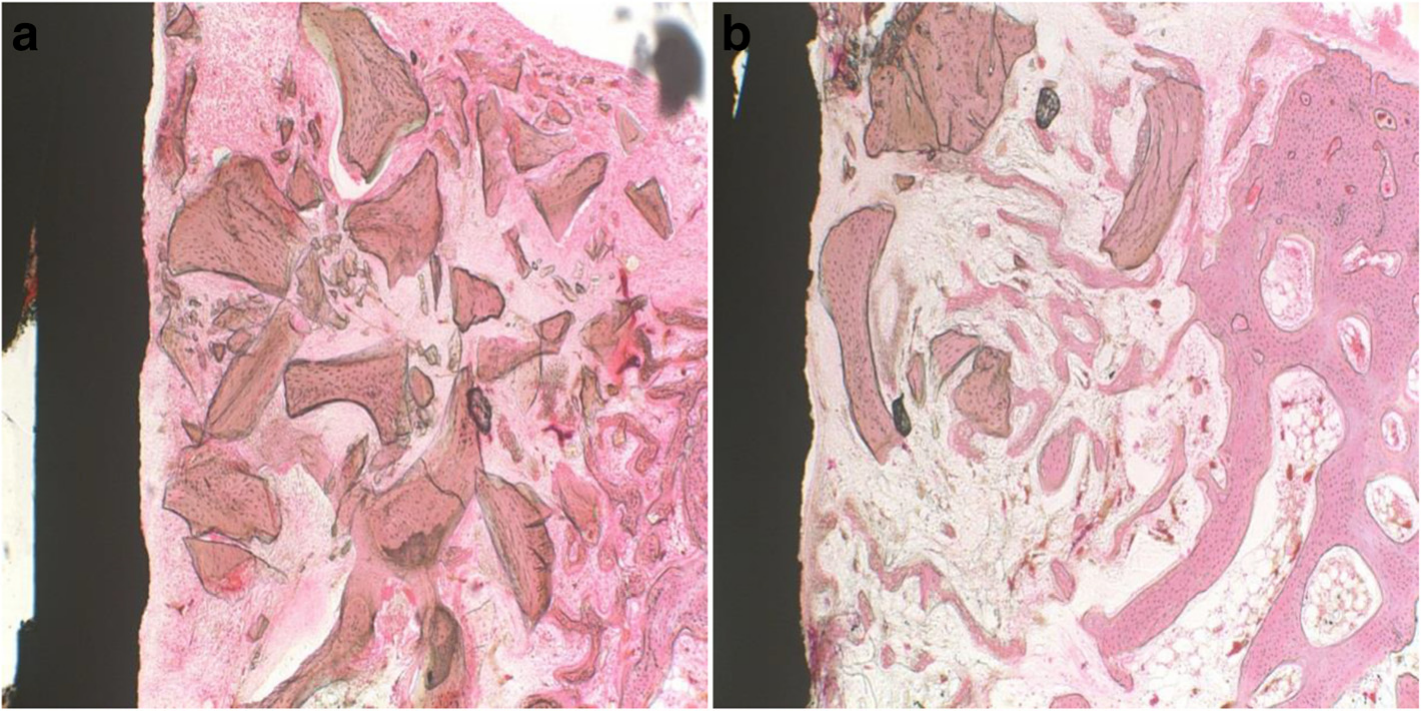
Histological sections at the second week. **a** Control group: defects treated with xenograft alone. **b** Experimental group: defects treated with xenograft and PRP. The new bone formation in the control group was observed more than the experimental group (2/3 vs. 1/3 of the gap, respectively). Original magnification ×100. H&E stain
- At 4 weeks. In the control group, the bone graft materials still remained. The newly formed bone, with low density, was in contact with the implant surface (Fig. 4a). In the experimental group, the trabecular bone was presented in 1/2 of the bone gap from the bone defect margin towards the implant surface and partially surrounded the bone graft materials, which were observed having a little amount of absorption (Fig. 4b).

Light microscopic examination of the sections showed the following:

- At 2 weeks. In the control group, the histological sections presented that bone graft materials were already starting to absorb, and the new bone formation was observed in 2/3 of the bone gap from the bone defect margin towards the implant surface (Fig. 3a). However, in experimental group, the new bone formation was only observed in 1/3 of the bone gap (Fig. 3b).

**Fig. 4:**
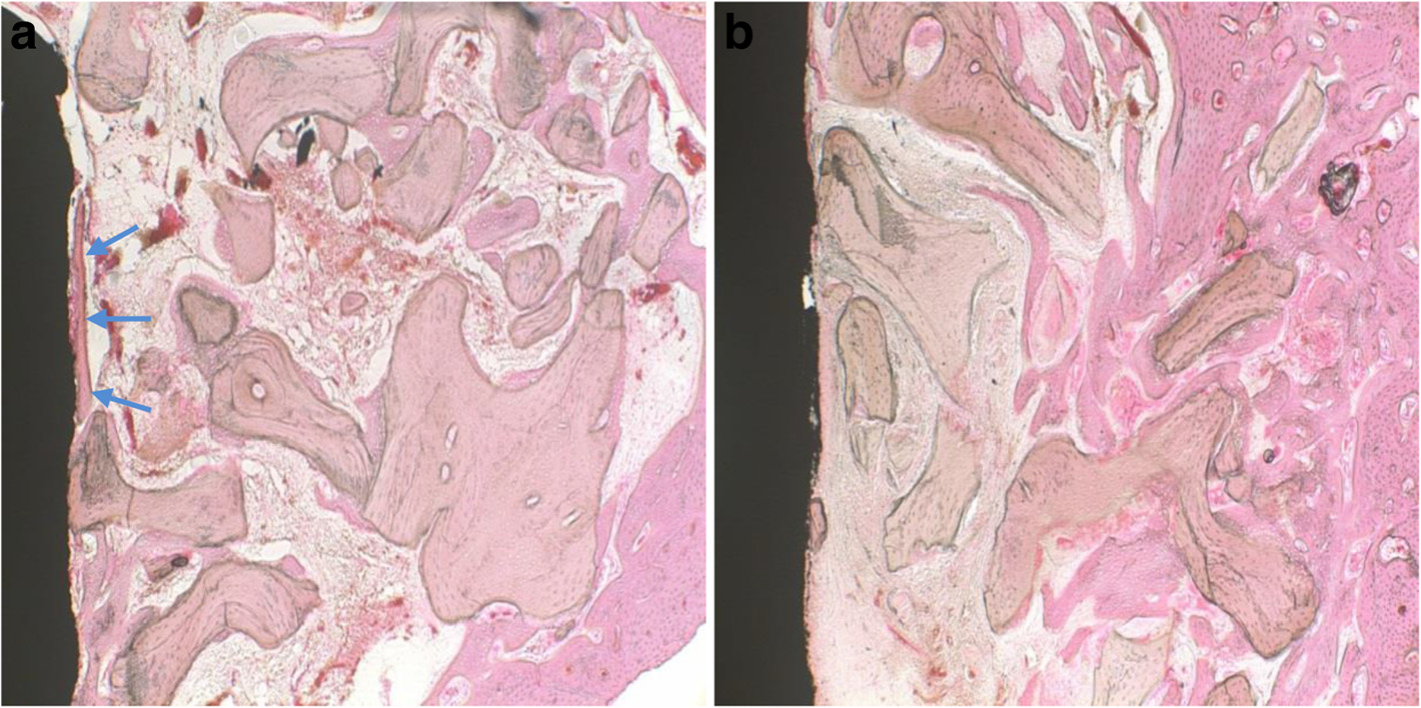
Histological sections at the fourth week. **a** Control group: defects treated with xenograft alone. **b** Experimental group: defects treated with xenograft and PRP. Newly formed bone can be observed in both groups. A limited bone-to-implant contact (*blue arrows*) was found in the control group. Original magnification ×100. H&E stain
- At 6 weeks. In the control group, the newly formed bone completely filled the bone gap and totally in contact with the implant surface. A small amount of residual bone graft materials was observed in the trabecular bone (Fig. 5a). In the experimental group, 2/3 of the bone gap was completely filled by the newly formed bone. The bone graft materials were considerably decreased than that in the fourth week (Fig. 5b).

**Fig. 5:**
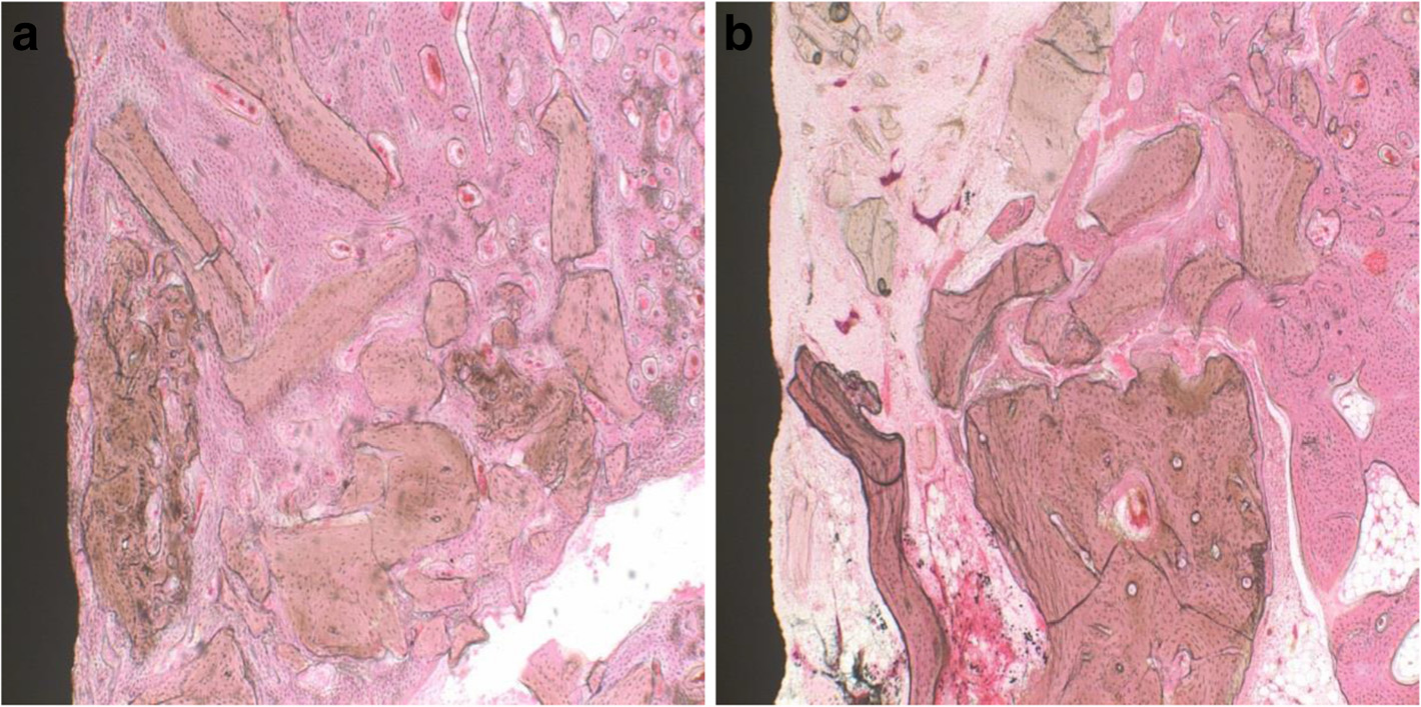
Histological sections at the sixth week. **a** Control group: defects treated with xenograft alone. **b** Experimental group: defects treated with xenograft and PRP. In the control group, the newly formed bone completely filled the bone gap; while in the experimental group, the newly formed bone filled 2/3 of the bone gap. Original magnification ×100. H&E stain

The defect areas of the rabbits in all groups showed various amounts of new bone formation; however, the defects of the animals of the experimental group generally contained the least amounts of new bone. Compared with the experimental group, the control group showed more newly formed bone and better bone healing process around the implants.

### Histomorphometric analysis

**Fig. 6:**
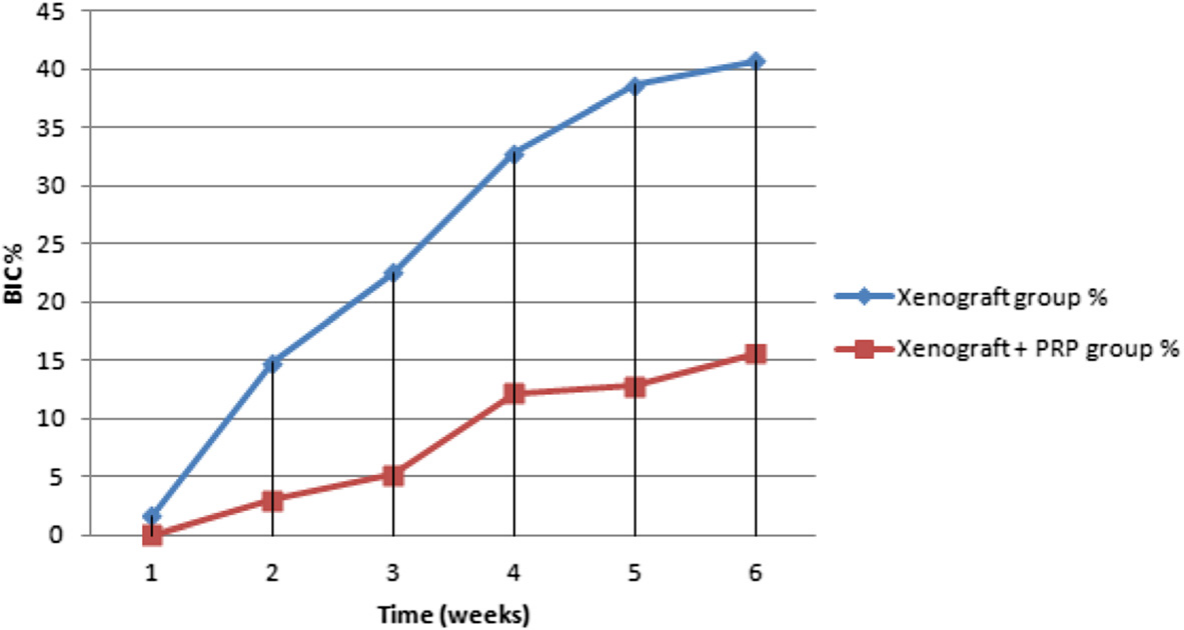
Mean percentage of bone-to-implant contact (BIC %) in rabbit’s tibia. the xenograft and PRP group shows lower BIC than the control group (statistically significant difference, *P* < 0.05)

The mean percentages of direct bone-to-implant contact in the two groups are shown in Table 1 and Fig. 6. The quantitative morphometric analysis showed significantly more bone-to-implant contact in the control group. The bone-to-implant contact was significantly higher (*P* < 0.05) in the control group (25.23 ± 15.15 %) than in the experimental group (8.16 ± 6.26 %).

**Table 1:** Mean percentage of bone-to-implant contact in rabbit’s tibia

Weeks	Xenograft group %	Xenograft + PRP group %
1	1.67	0
2	14.88	3.01
3	22.59	5.23
4	32.81	12.19
5	38.69	12.88
6	40.71	15.62
Mean ± SD	25.23 ± 15.15*	8.16 ± 6.26*

*Statistically significant difference, *P* < 0.05

## Discussion

Although autogenous grafts are commonly used in oral and maxillofacial surgery for treatment of bone loss and functional rehabilitation, the need for additional intervention increases the duration of surgery and the risk of infection, pain, and discomfort at the donor site. During the last decade, several bone grafting materials produced from bovine bone, with physicochemical characteristics similar to those of human bone, have been developed for use in oral and orthopedic surgeries as an alternative to autogenous grafts [13]. In a study on rabbit, Jensen et al. [14] found that Bio-Oss became completely incorporated in newly formed bone. In comparison with other three bone substitute materials (ceramic hydroxyapatite, coralline hydroxylapatite, and coral calcium carbonate), Bio-Oss showed a higher degree of integration in the surrounding bone. Berglundh and Lindhe [15] also concluded that Bio-Oss became integrated with the host bone and subsequently replaced by newly formed bone. Bio-Oss contains pores with different sizes, intracrystalline spaces (3–26 nm), micro pores (vascular marrow canals), and macro pores (300–1500 μm), which result in a high overall porosity of 70–75 % [16]. As a consequence of this highly porous structure, Bio-Oss can be easily invaded by blood vessels resulting in subsequent migration of osteoblasts. Therefore, Bio-Oss is considered to be a biocompatible grafting material with remarkable osteoconductive ability, which does not cause significant inflammatory reaction [17].

Some authors such as Marx et al., Magesh et al. [18], and Aimetti et al. [19] evaluated the effect of PRP on bone regeneration in human. In their studies, the bone defects were treated with an autogenous bone graft alone or in combination with PRP, and they all demonstrated that the use of PRP along with an autogenous bone graft were advantageous since it enhanced the quantity of newly formed bone. Yilmaz et al. [20] investigated the effectiveness of PRP and a bovine-derived xenograft (BDX) combination on early wound healing in deep intrabony defects. A total of 85 intrabony defects were selected in 20 advanced chronic periodontitis patients. Defects were surgically treated with PRP/BDX. One year after surgery, the results showed that PRP in combination with BDX leads to a significantly favorable clinical improvement in deep intrabony periodontal defects.

Nagata et al. [21] analyzed the effect of PRP on healing of autogenous bone (AB) grafts placed in surgically created critical-size defects in rabbit calvaria. The results indicated that AB/PRP significantly improved bone formation, and a beneficial effect of PRP was limited to an initial healing period of 4 weeks. In the study of Kurikchy et al. [22], they assessed the effect of PRP on the bone healing process either alone or mixed with xenogenic graft (Gen-Ox-lyophilized bovine bone organic matrix) in the femur bone defects of rabbit models. The results showed that in the use of PRP in combination with the xenogenic bone graft, new bone formation and neovascularization were enhanced significantly when compared with xenogenic graft alone.

Furthermore, Torres et al. [23] evaluated the clinical efficacy of PRP in a sinus augmentation procedure with implant placement. Eighty-seven patients underwent 144 sinus floor augmentation procedures using anorganic bovine bone (ABB) alone or ABB + PRP. A total of 286 implants were placed in the augmented bone. After a follow-up period of 24 months, the histological analysis in the five edentulous patients revealed that bone augmentation was significantly higher in sites treated with ABB + PRP (*P* < 0.05). Cho et al. [24] investigated the effect of PRP on dental implant osseointegration. Sixteen dental implants 4 mm in diameter and 8 mm in length were placed into each tibia of four dogs. The experimental groups were treated with 0.5 mL PRP; the control groups were instilled with 0.5 mL of saline. Four weeks after implantation, the experimental group showed significantly faster bone regeneration and increased bone activity compared to the control group (*P* < 0.05).

However, there are different opinions regarding the effect of PRP on bone formation. In a study on mini-pigs, Wiltfang et al. [25] investigated the influence of PRP on the regeneration of bony defects in the forehead region. The defects were filled with randomly distributed combinations of autogenous bone and xenogenic bone substitutes (Bio-Oss) with and without PRP. After 12 weeks of healing periods, microradiographic results showed a significant effect on bone regeneration in the autogenous group when PRP is added; however, when using xenogenic bone substitutes, PRP did not display favorable results and may even cause adverse effects. Also, in an experimental study reported by Thorwarth et al. [26], the defects in the frontal skull of domestic pigs were filled with deproteinized bovine bone matrix (DBBM) or autogenous bone with or without PRP. The microradiographic evaluation demonstrated a statistically significant enhancement in bone regeneration by PRP only after use of autogenous bone plus PRP. However, in all DBBM groups, bone formation remained unchanged, and no effects of the PRP administration were found in the mineralization process, demonstrating the lack of osteoinductive capacity in PRP.

In a clinical study, Schaaf et al. [27] examined the influence of PRP on autogenous bone grafting in sinus floor augmentation. Thirty-four patients undergoing sinus augmentation before implant placement and the experimental group had additional treatment with PRP. Four months later, radiographic imaging analysis showed that there was no significantly increased bone density when PRP was used in combination with autogenous bone grafting compared with autogenous bone alone. Cabbar et al. [28] compared the effect of PRP with or without xenograft (Unilab Surgibone) to augment the human maxillary sinus. In the experimental group, sinuses on one side were filled with xenograft and PRP combination, whereas in the control group, sinuses on the opposite side were filled with xenograft alone. After a mean period of 6.8 months, histological analysis showed that the volumes of soft tissue were 57.8 ± 4.4 % and 59.9 ± 7.5 % in the experimental and control groups, respectively; residual grafting materials were 23.6 ± 5.9 % and 21.9 ± 6.6 %, respectively; and new bone were 16.0 ± 3.8 % and 15.8 ± 4.8 %, respectively. There were no statistically significant differences found between the groups (*P* > 0.05).

Hatakeyama et al. [10] and Choi et al. [29] reported the effect of PRP on bone regeneration in autogenous bone grafts by using rabbit and dog models, respectively. According to their results, they both suggested that the addition of PRP did not enhance new bone formation in autogenous bone grafts.

Moreover, Froum et al. [30] presented a clinical report of three patients undergoing sinus floor augmentation treated with PRP + anorganic bovine bone (experimental group) or anorganic bovine bone alone (control group). Miniature test implants, 2.0 mm in diameter and 10 mm in length, were placed through the crestal bone into the sinus grafts. Histomorphometric analysis indicated that the addition of PRP to the grafts did not make a significant difference either in vital bone production or in interfacial bone contact on the test implants. Sánchez et al. [31] investigated whether the addition of PRP to xenogeneic bone grafts (demineralized freeze-dried bone graft) would increase the rate of bone formation. Ninety dental implants were inserted in the mandibles of nine dogs; subsequently, three-wall peri-implant defects were surgically created. Defects were randomly assigned to three groups: PRP + xenograft, xenograft alone, and no treatment. No differences were observed in bone formation among the three groups.

In our experiment, a defect between the bony wall and the implant surface in rabbit tibia was made, and then treated with bovine-derived xenograft with or without PRP. As the result, a better bone healing process and more amount of new bone formation were observed in the control group. The percentage of bone-to-implant contact in the control group was 25.23 ± 15.15 %, whereas in the experimental group, the percentage was only 8.16 ± 6.26 %. According to the results of histological and histomorphometric examinations, our study is in agreement with the findings from previous studies in which there was no effect of PRP on new bone formation in the PRP-treated bone graft.

It is not very clear why the PRP-treated grafts exhibited decreased bone formation when compared with the non-PRP-treated grafts. Between and within the species, the baseline values of the platelet numbers have a great variation [32]. This variation of the platelet concentration may have an important effect in the conflicting results reported in various animal experimental studies using PRP. Regarding several studies carried out in humans indicating an advantageous effect of PRP, it may be possible that human PRP is more potent than animal-derived PRP. It should be born in mind that these human studies are designed for comprehensible reasons, but without randomized prospective, have no control sites and comprise a heterogeneous group of patients. A recently performed animal experiment by Plachokova et al. [33] supports this suggestion. In their experiment, they compared the bone regenerative effect of PRPs of different species (rat, goat, and human). PRPs in combination with human bone or HA/TCP (hydroxyapatite-tricalcium phosphate) were used in nude rat models with critical-size cranial defects. As a result, no effect of rat PRP and goat PRP was seen, while human PRP mixed with human bone significantly enhanced new bone formation, but only after 2 weeks postoperatively. The authors noted that, in comparison with the preparation of human PRP, the method of animal PRP preparation should be changed for different animal species. The authors also noted the importance of defining the different critical effective amounts of platelet and growth factor levels in PRP according to different animal species. For this reason, the critical effective amount of platelets in each type of animal should be defined by experimental studies.

Another factor may be related to the concentration of PRP within the grafts. When a small amount of bone graft is mixed with a large volume of PRP, the activation of bone cells in the adjacent tissue or graft and enhancement of new bone formation would be expected. Weibrich et al. [34] analyzed the effect of the platelet count in PRP on bone regeneration. In their study, three types of platelet concentrations in PRP were used in the rabbit models, including low-platelet concentrations (164,000–373,000 platelets/μL), intermediate platelet concentrations (503,000–1,729,000 platelets/μL), and high platelet concentrations (1,845,000–3,200,000 platelets/μL). Comparing the bone regeneration after 4 weeks, the only slightly significant difference was seen with intermediate platelet concentrations (*P* = 0.004), but in analyzing the bone-to-implant contact, no differences were found for the three platelet concentration groups. In our study, the baseline value of platelets in the whole blood was 236 × 10^3^/μL, whereas the platelet concentration of PRP was 625 × 10^3^/μL. Thus, a 265 % increase in platelet count was observed. However, the results failed to show an increase in bone-to-implant contact when this concentration of PRP was used.

PRP is commonly used in different clinical situations in an attempt to improve soft and hard tissue healing. In spite of this, the results from our study showed that the addition of PRP to bovine-derived xenograft in the defects around the implants in the present animal model did not result in increased new bone formation or bone-to-implant contacts. More basic researches into animal species, optimal concentration of PRP, grafting materials, and presence of implants are necessary to capitalize on the ability of platelet growth factors to enhance bone formation in a graft.

## Conclusions

This study reported the healing effect of PRP on bovine-derived xenograft in peri-implant defects. Intrabony defects were created in the tibia of rabbits, and dental implants were installed in defects filled with either xenograft alone or mixture of PRP and xenograft. The mean percentage of bone-to-implant contact in the defects treated with the xenograft alone was 25.23 ± 15.15 %, whereas in the defects treated with xenograft combined with PRP, the percentage was only 8.16 ± 6.26 %. On the basis of these findings, it can be concluded that the addition of PRP into bovine-derived xenograft around titanium dental implants may delay peri-implant bone healing. So far, the scientific evidence regarding the efficacy and efficiency of PRP is still controversial.

### Statement of ethics approval

Animal protocol was approved by INHA University, Institutional Animal Care, and the approval number is INHA 150716-372.
